# Adaptation of SARS-CoV-2 in BALB/c mice for testing vaccine efficacy

**DOI:** 10.1126/science.abc4730

**Published:** 2020-07-30

**Authors:** Hongjing Gu, Qi Chen, Guan Yang, Lei He, Hang Fan, Yong-Qiang Deng, Yanxiao Wang, Yue Teng, Zhongpeng Zhao, Yujun Cui, Yuchang Li, Xiao-Feng Li, Jiangfan Li, Na-Na Zhang, Xiaolan Yang, Shaolong Chen, Yan Guo, Guangyu Zhao, Xiliang Wang, De-Yan Luo, Hui Wang, Xiao Yang, Yan Li, Gencheng Han, Yuxian He, Xiaojun Zhou, Shusheng Geng, Xiaoli Sheng, Shibo Jiang, Shihui Sun, Cheng-Feng Qin, Yusen Zhou

**Affiliations:** 1 State Key Laboratory of Pathogen and Biosecurity, Beijing Institute of Microbiology and Epidemiology, Academy of Military Medical Sciences, Beijing 100071, China.; 2 State Key Laboratory of Proteomics, Beijing Proteome Research Center, National Center for Protein Sciences (Beijing), Beijing Institute of Lifeomics, Beijing 102206, China.; 3 Institute of Military Cognition and Brain Sciences, Beijing 100850, China.; 4 Institute of Pathogen Biology, Chinese Academy of Medical Sciences and Peking Union Medical College, Beijing 100730, China.; 5 Laboratory Animal Center, Academy of Military Medical Sciences, Beijing 100071, China.; 6 Beijing JOINN Biologics Co., Beijing 100176, China.; 7 Key Laboratory of Medical Molecular Virology (MOE/NHC/CAMS), School of Basic Medical Sciences, Fudan University, Shanghai 200032, China.

## Abstract

The ongoing coronavirus disease 2019 (COVID-19) pandemic has prioritized the development of small-animal models for severe acute respiratory syndrome coronavirus 2 (SARS-CoV-2). We adapted a clinical isolate of SARS-CoV-2 by serial passaging in the respiratory tract of aged BALB/c mice. The resulting mouse-adapted strain at passage 6 (called MASCp6) showed increased infectivity in mouse lung and led to interstitial pneumonia and inflammatory responses in both young and aged mice after intranasal inoculation. Deep sequencing revealed a panel of adaptive mutations potentially associated with the increased virulence. In particular, the N501Y mutation is located at the receptor binding domain (RBD) of the spike protein. The protective efficacy of a recombinant RBD vaccine candidate was validated by using this model. Thus, this mouse-adapted strain and associated challenge model should be of value in evaluating vaccines and antivirals against SARS-CoV-2.

## Modeling SARS-CoV-2 in mice

Among the research tools necessary to develop medical interventions to treat severe acute respiratory syndrome coronavirus 2 (SARS-CoV-2) infections, high on the list are informative animal models with which to study viral pathogenesis. Gu *et al.* developed a mouse model in which a SARS-CoV-2 strain was infectious and could cause an inflammatory response and moderate pneumonia. Adaptation of this viral strain in the mouse appeared to be dependent on a critical amino acid change, Asn^501^ to Tyr (N501Y), within the receptor-binding domain of the viral spike protein. The new mouse model was used to study neutralizing antibodies and a vaccine candidate against the virus.

*Science*, this issue p. 1603

## Teaser

A SARS-CoV-2 mouse model is used to study viral responses and the development of vaccine candidates.

The pandemic of coronavirus disease 2019 (COVID-19) caused by the newly emerged severe acute respiratory syndrome coronavirus 2 (SARS-CoV-2) has become a global health crisis (*1*–*3*). In the absence of protective immunity in the whole human population (*4*), SARS-CoV-2 has exhibited an unprecedented human-to-human transmission capability. Although several vaccine candidates are being currently tested in clinical trials, no commercial COVID-19 vaccine is presently available.

SARS-CoV-2 belongs to the *Betacoronavirus* genus of the *Coronaviridae* family, along with two other closely related highly pathogenic viruses, SARS-CoV and Middle East respiratory syndrome coronavirus (MERS-CoV). SARS-CoV-2 has a positive-sense, single-stranded RNA genome of 30 kb in length, which is coated by the inner nucleocapsid (N) proteins and an outer envelope made up of membrane (M) and envelope (E) proteins, as well as spike (S) proteins. Like SARS-CoV, the S protein of SARS-CoV-2 mediates viral entry into host cells by binding to their shared receptor, angiotensin-converting enzyme 2 (ACE2), through the receptor-binding domain (RBD) (*1*). Previously, we and others have demonstrated that the RBD of SARS-CoV and MERS-CoV contain major conformation-dependent neutralizing epitopes and are capable of eliciting potent neutralizing antibodies in immunized animals, thus representing promising targets for vaccine development (*5*–*8*).

Small-animal models that recapitulate SARS-CoV-2 infection are urgently needed. Because SARS-CoV-2 does not use mouse ACE2 as its receptor (*1*), wild-type mice are thought to be less susceptible to SARS-CoV-2. Transgenic mice expressing human ACE2 have been developed by means of different strategies. Such mice have been used previously to study SARS-CoV-2 infection and pathogenesis and to evaluate countermeasures against COVID-19 (*9*–*11*). Here, we report the generation of a mouse-adapted strain of SARS-CoV-2 that can productively replicate in the respiratory tract and cause interstitial pneumonia in wild-type immunocompetent mice. Additionally, the protective efficacy of a newly developed recombinant subunit vaccine candidate based on SARS-CoV-2 RBD was assayed by using this mouse challenge model.

## Results

### Rapid adaption of SARS-CoV-2 in BALB/c mice

**Fig. 1:**
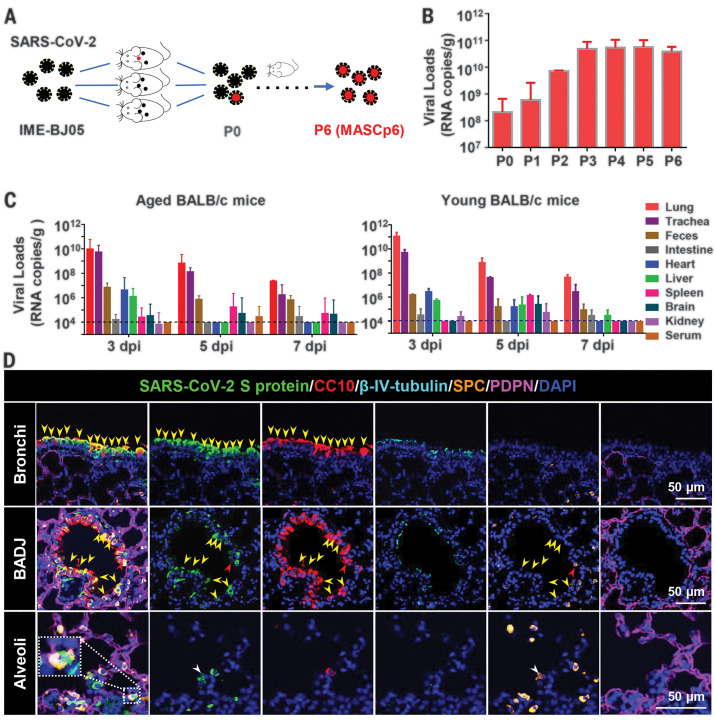
Generation and characterization of a mouse-adapted strain of SARS-CoV-2 in BALB/c mice. (**A**) Schematic diagram of the passage history of SARS-CoV-2 in BALB/c mice. The original SARS-CoV-2 viruses are shown in black, and the adapted viruses are in red. (**B**) SARS-CoV-2 genomic RNA loads in mouse lung homogenates at P0 to P6. Viral RNA copies were determined by means of quantitative reverse transcription polymerase chain reaction (RT-PCR). Data are presented as means ± SEM (*n* = 2 to 4 mice per group). (**C**) Tissue distribution of SARS-CoV-2 viral RNAs in mice infected with MASCp6. Groups of aged and young mice were inoculated with 1.6 × 10^4^ PFU of MASCp6 and sacrificed at 3, 5, or 7 days after inoculation, respectively. Feces, sera, and the indicated tissue samples were collected at the specified times and subjected to viral RNA load analysis by means of quantitative RT-PCR. Dashed lines denote the detection limit. Data are presented as means ± SEM (*n* = 3 mice per group). (**D**) Multiplex immunofluorescence staining of mouse lung sections. SARS-CoV-2 S protein (green), CC10 (red), β-IV-tubulin (cyan), PDPN (magenta), SPC (gold), and nuclei (blue). The dash box is magnified at the bottom right corner of the same image. Yellow arrowheads indicate SARS-CoV-2^+^/CC10^+^ cells, redarrow heads indicate SARS-CoV-2^+^/CC10^+^/SPC^+^ cells, and the white arrowheads indicate SARS-CoV-2^+^/SPC^+^ cells.

To generate a SARS-CoV-2 mouse-adapted strain, the human clinical isolate of SARS-CoV-2 (BetaCov/human/CHN/Beijing_IME-BJ05/2020, abbreviated as IME-BJ05) was serially passaged by means of intranasal inoculation in aged mice (Fig. 1A), as previously described for SARS-CoV (*12*). Briefly, 9-month-old BALB/c mice were intranasally inoculated with 7.2 × 10^5^ plaque forming units (PFU) of SARS-CoV-2, and the lung tissues were collected from each passage for viral RNA load analysis at 3 days after inoculation. Substantial viral RNAs (10^8.32^ copies/g) were readily detected after a single passage, which was defined as passage 0 (P0), in the lung homogenate (Fig. 1B). Subsequently, the viral RNA copies in the lung approached 10^10.68^ RNA copies/g at passage 3 (P3), which was about 250-fold higher than those at P0 and remained at a similar level during the following passages (Fig. 1B). The final viral stock at passage 6 (P6) was titrated by means of plaque assays (fig. S1A) and called MASCp6 for further characterization.

To determine whether the increased viral RNA loads in mouse lungs could be attributed to the enhanced infectivity of the virus in mice, we examined the replication kinetics and tissue tropism of MASCp6 in both aged (9 months old) and young (6 weeks old) BALB/c mice. After intranasal inoculation with 1.6 × 10^4^ PFU of MASCp6, high amounts of viral RNAs in the lungs and tracheas were detected at 3, 5 and 7 days after inoculation in all aged mice (Fig. 1C), with peak viral RNA loads of ~10^10^ copies/g at 3 days after inoculation, which was comparable with the results from the human ACE2 transgenic mice (*10*). Viral RNAs were also detected in heart, liver, spleen, and brain, as well as in feces. Marginal viral RNA was detected in the kidney and serum from individual infected mice (Fig. 1C). Similar tissue distribution of SARS-CoV-2 RNA was also seen in the MASCp6-infected young mice (Fig. 1C). Immunostaining of lung section from MASCp6-infected mice showed robust expression of S protein along the airways and at the alveolus in both young and aged mice at 3 and 5 days after inoculation (fig. S1B). To identify the major cell types infected by SARS-CoV-2 in our model, lung sections were further analyzed by means of multiplex immunofluorescence staining for SARS-CoV-2 S protein and specific lung epithelium cell markers. As shown in Fig. 1D, colocalization of CC10^+^ club cells and SARS-CoV-2 S protein were observed predominantly in the bronchi and bronchioles as well as the bronchioalveolar-duct junction (BADJ) of the lungs. Furthermore, SPC^+^ alveolar type 2 (AT2) cells were also costained with S protein in the BADJ and alveoli. However, SARS-CoV-2 S protein was not detected in all β-IV-tubulin^+^ ciliated cells and PDPN^+^ alveolar type 1 (AT1) cells. Thus, club cells and AT2 cells are the major target cells that support SARS-CoV-2 replication in mouse lung in our model.

### Characterization of MASCp6 infection in BALB/c mice

**Fig. 2:**
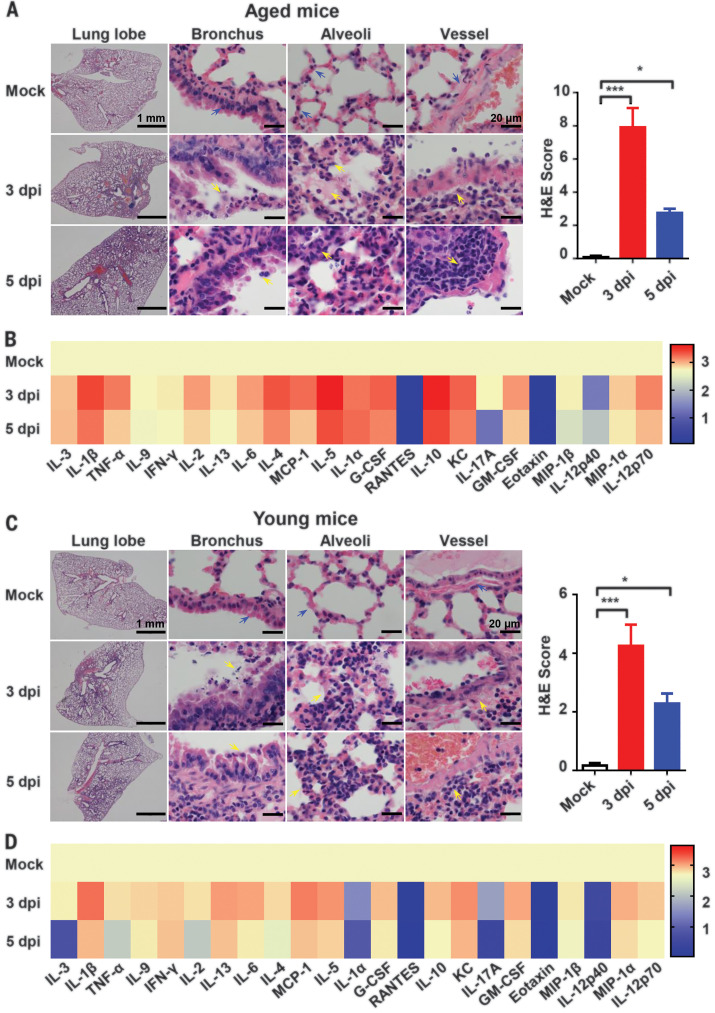
MASCp6 infection causes pathological lung lesions and inflammatory responses in both aged and young BALB/c mice. (**A**) H&E staining of lung sections from aged (9 months old) BALB/c mice infected with MASCp6. Blue arrows indicate the normal areas, and yellow arrows indicate damaged areas. Data from semiquantitative analysis of histopathological changes of lung tissues are presented as means ± SEM (*n* = 3 mice per group). Statistical significance was analyzed by means of Mann-Whitney test. (**B**) Serum cytokine and chemokine heatmap in MASCp6-infected aged mice. Data are presented as fold change relative to mock infection (*n* = 5 mice per group). (**C**) H&E staining of lung sections from MASCp6-infected young mice (*n* = 3 mice per group). (**D**) Serum cytokine and chemokine heatmap in MASCp6-infected young mice (*n* = 5 mice per group). *P < 0.05, ***P < 0.001.

To further characterize pathological features in the MASCp6-infected BALB/c mice, lung tissues were collected at 3 or 5 days after inoculation, respectively, and subjected to histopathological analysis by means of hematoxylin and eosin (H&E) staining. Both aged and young BALB/c mice presented with mild to moderate pneumonia after MASCp6 infection (Fig. 2, A and C). In the aged mice, MASCp6 infection caused interstitial pneumonia at 3 days after inoculation, characterized with denatured and collapsed epithelial cells, thickened alveolar septa, alveolar damage, focal exudation and hemorrhage, and activated inflammatory cell infiltration. Vessels were obviously injured, with adherent inflammatory cells and damaged basement membrane (Fig. 2A). At 5 days after inoculation, the lung damage was much milder than that seen at 3 days after inoculation (Fig. 2A), suggesting a self-recovering process. In addition, multiplex immunofluorescence staining demonstrated that SARS-CoV-2 infection led to massive cell death, as evidenced with cleaved caspase-3 staining, at 3 days after inoculation, as well as remarkable inflammatory cell infiltration in CD103^+^ dendritic cells, CD163^+^ macrophages, and CD3^+^ T lymphocytes in the lung of aged mice (fig. S2A). Serum concentrations of inflammatory cytokines—including interleukin-1β (IL-1β), IL-6, and IL-5—were up-regulated upon MASCp6 challenge (Fig. 2B and fig. S3A). As expected, the young mice developed similar but much milder lung damage than that of the aged mice after MASCp6 challenge (Fig. 2C). Similarly, inflammatory cell infiltration (fig. S2B) and serum cytokine response (Fig. 2D and fig.S3B) in the young mice was much weaker than that of aged mice. Additionally, MASCp6 infection caused no obvious changes in the body weight of aged or young mice (fig. S4). Taken together, these results demonstrate that MASCp6 can productively replicate in the lower respiratory tract of wild-type BALB/c mice, resulting in a more severe interstitial pneumonia phenotype in the aged mice.

### Identification of adaptive mutations that emerged in MASCp6

**Fig. 3:**
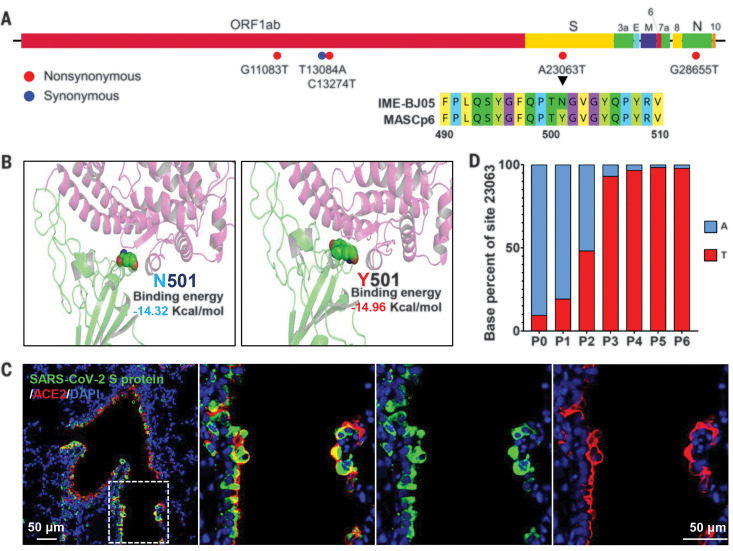
MASCp6 carries a distinct amino acid substitution in the RBD of the Spike (S) protein. (**A**) Schematic diagram of SARS-CoV-2 genome and all the adaptive mutations identified in MASCp6. Amino acid sequences of the parental IME-BJ05 strain and the MASCp6 strain adjacent to the N501Y mutation are shown. (**B**) Homology modeling of mouse ACE2 (pink) in complex with SARS-CoV-2 RBD (green) with N501 (left) or Y501 residue (right). (**C**) Colocalization of SARS-CoV-2 S protein (green) and mouse ACE2 (red) in the lung from SARS-CoV-2–infected mice. The dashed box in the left image is magnified in the three images at the right. (**D**) The proportion of A23063T mutation in each passage. The mutation threshold was defined as 1% according to the average quality score of sequenced base.

To decipher the underlying mechanism for the increased virulence of MASCp6, the complete genome of MASCp6 was subjected to deep sequencing with an Ion Torrent S5Plus sequencer. Compared with the full genome of the original SARS-CoV-2 strain IME-BJ05, MASCp6 contains five nucleotide mutations that are distributed within the ORF1ab, S, and N genes, respectively (Fig. 3A and table S1). The A23063T mutation resulted in a N501Y amino acid substitution in the RBD of the S protein, which is assumed to be responsible for receptor recognition and host range of SARS-CoV-2 (*13*, *14*). (A, alanine; T, threonine; N, asparagine; Y, tyrosine. In the mutants, other amino acids were substituted at certain locations; for example, A23063T indicates that alanine at position 23063 was replaced by threonine.) Structural remodeling also suggested that the N501Y substitution in the RBD of SARS-CoV S protein increased the binding affinity of the protein to mouse ACE2 (Fig. 3B). Immunofluorescence staining supported colocalization of mouse ACE2 and SARS-CoV-2 S protein in the lungs of MASCp6-infected mice (Fig. 3C). To further trace the adaption history of MASCp6, the emergence of N501Y substitution was further analyzed by means of deep sequencing. As expected, all reads from the original IME-BJ05 isolate were pure A23063. T23063 readily emerged after a single passage in one of the three mouse lung homogenates (table S2), and the proportion of A23063T mutation gradually increased during subsequent passages (Fig. 3D). Thus, the increased virulence of SARS-CoV-2 MASCp6 in mice was likely attributed to the rapid emergence of N501Y substitution in the RBD of SARS-CoV-2 S protein.

### Validation of the protective efficacy of an RBD-based SARS-CoV-2 subunit vaccine

To validate the utility of this mouse challenge model, we tested the protection efficacy of a recombinant subunit vaccine candidate against COVID-19. Briefly, SARS-CoV-2 RBD (aa 331-524) fused with a human immunoglobulin G (IgG) Fc at the C terminus (fig. S5A) was expressed in CHO-K1 cells and purified through affinity chromatography and anion exchange chromatography in a good laboratory practice (GLP) laboratory. As expected, the molecular weight of recombinant RBD-Fc was about 47.98 kD, as detected with mass spectroscopy (fig. S4B), and flow cytometry analysis confirmed that RBD-Fc, not the Fc control, specifically bound to human ACE2 expressed in the stable ACE2/293T cells (fig. S4C) (*15*).

**Fig. 4:**
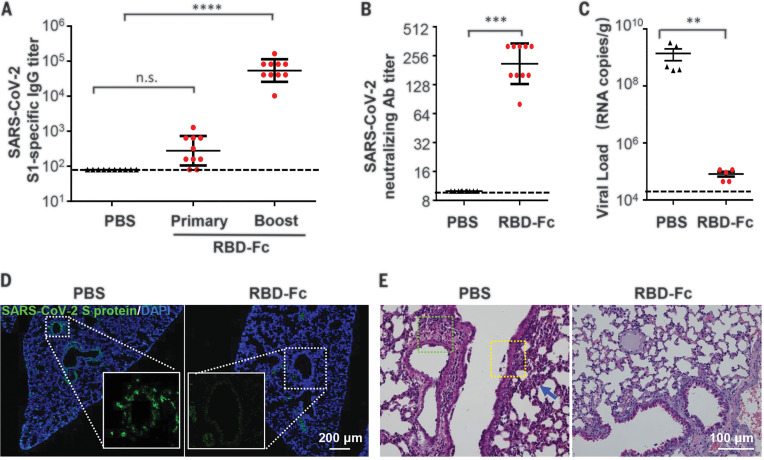
Protection efficacy of the recombinant RBD-Fc vaccine candidate against MASCp6 challenge in mice. (**A**) SARS-CoV-2–specific IgG antibody titers were detected with enzyme-linked immunosorbent assay at 2 weeks after primary and boost immunization, respectively (*n* = 10 mice per group). Statistical significance was analyzed by means of one-way analysis of variance. (**B**) Neutralizing antibody titers against SARS-CoV-2 were determined with the microneutralization assay at 2 weeks after boost immunization (*n* = 10 mice per group). (**C**) Viral RNA loads in lung of vaccinated mice were detected at 5 days after MASCp6 challenge (*n* = 5 mice per group). Statistical significance was analyzed by means of Student’s *t* test. (**D**) Immunofluorescence staining of mouse lung sections for S protein (green) and 4′,6-diamidino-2-phenylindole (DAPI) (blue). The dotted boxes are magnified at the bottom of the same image. (**E**) H&E staining of mouse lung sections. Focal perivascular (green square) and peribronchiolar (yellow square) inflammation and thickened alveolar septa (blue arrow) are indicated. n.s., not significant; **P < 0.01, ***P < 0.001, ****P < 0.0001.

Female BALB/c mice were then subcutaneously immunized with two doses of recombinant RBD-Fc (10 μg/mouse) at a 2-week interval, and mice immunized with phosphate-buffered saline (PBS) were set as controls. As expected, high levels of SARS-CoV-2–specific IgG antibodies (Fig. 4A) and neutralization antibodies (Fig. 4B) were elicited in all of the RBD-Fc immunized mice at 2 weeks after boost immunization. All immunized mice were then intranasally challenged with MASCp6 (1.6 × 10^4^ PFU), and lung tissues were collected for virological and histopathological analysis at 5 days after challenge. As expected, all the PBS-treated mice sustained high amounts of viral RNA loads in the lung at 5 days after challenge. By contrast, a significant reduction in viral RNA loads (approximately 0.1%) were seen in the lung of RBD-Fc immunized mice compared with the control animals (Fig. 4C). Moreover, immunofluorescence staining for SARS-CoV-2 S protein showed that only a small population of positive cells was detected in the lung from the RBD-Fc–immunized mice, whereas abundant viral proteins were seen in the lung from PBS-immunized mice (Fig. 4D). No apparent pathological damage was observed in the lung of RBD-Fc immunized mice, whereas inflammatory lung injury—with focal perivascular and peribronchiolar inflammation, as well as thickened alveolar septa—were found in the lung of the control mice (Fig. 4E). Taken together, these data indicate that our newly developed mouse model with MASCp6 represents a useful tool for testing the efficacy of COVID-19 vaccine candidates.

## Discussion

An ideal animal model for COVID-19 should reproduce the viral replication as well as the clinical outcome observed in COVID-19 patients. Here, we report the rapid adaption of SARS-CoV-2 in BALB/c mice, and the resulting MASCp6 strain not only replicated efficiently in the trachea and lung but also caused interstitial pneumonia and inflammatory responses, reproducing many clinical features observed in COVID-19 patients (*16*, *17*). Upon MASCp6 challenge, SARS-CoV-2 primarily replicated in the respiratory tracts, and viral RNAs peaked in the lungs at 3 days after inoculation and then decayed at 5 and 7 days after inoculation. This result was consistent with other transgenic or humanized mouse models (*9*, *11*). In particular, the aged mice developed more severe lung damage when compared with the young mice upon MASCp6 challenge, which reflects that the mortality and fatality of COVID-19 are strongly skewed toward the elderly (*18*). Fatality was only reported by Jiang *et al*. (*10*) in SARS-CoV-2–infected ACE2 transgenic mice. In our challenge model, neither visible clinical symptoms nor body weight loss were recorded throughout the experiments (fig. S4). The challenge dose used in our experiment was 1.6 × 10^4^ PFU; thus, whether a higher challenge dose of MASCp6 would exacerbate the pathology remains to be determined.

The development of a mouse-adapted strain-based challenge model has been well demonstrated in SARS-CoV and MERS-CoV studies (*12*, *19*, *20*). Serial passaging of virus in mouse lungs results in adaptive mutations that increase viral infectivity. The MASCp6 genome contains five mutations in comparison with its parental strain IME-BJ05, and these mutations resulted in four amino acid residue changes in the ORF1ab, S, and N genes, respectively (Fig. 3A). The N501Y mutation seems to provide a more favorable interaction with mouse ACE2 for docking and entry, thus leading to the increased virulence phenotype in mice. Whether the other three mutations, except for N501Y, also regulated viral infectivity remains to be determined. Further investigation with reverse genetics will clarify this issue and could allow the rapid synthesis of a recombinant SARS-CoV-2 with enhanced virulence (*21*, *22*). Additionally, immunostaining results showed that lung club and AT2 cells are major target cells of MASCp6, which is in agreement with previous findings from animal models and COVID-19 patients (*11*, *23*, *24*).

Compared with the previously described ACE2 transgenic or humanized mice, our MASCp6-based challenge model uses immunocompetent wild-type mice and can be directly applied to the efficacy evaluation of various vaccine candidates. Immunization with the recombinant subunit vaccine candidate (RBD-Fc) induced high levels (up to 1:320) of neutralizing antibodies against SARS-CoV-2, nearly eliminating viral RNA replication in mouse lungs after MASCp6 challenge (Fig. 4, B and C). The potential correlation between serum neutralizing antibody titers in the vaccinated mice and the protective efficacy highlights the versatility of this convenient and economical animal model. Recently, nonhuman primates, which are closest to humans phylogenetically, have also been used to reproduce SARS-CoV-2 infection, and several vaccine candidates have been validated with promising protection efficacy (*25*–*27*). Hamsters, ferrets, and cats are also permissive to SARS-CoV-2 infection (*28*–*30*), and the clinical outcome varies from asymptomatic infection to severe pathological lung lesions after SARS-CoV-2 infection. No single animal model for SARS-CoV-2 currently reproduces all aspects of the human disease. Therefore, the establishment of different animal models should greatly expand our understanding of SARS-CoV-2 transmission and pathogenesis and accelerate the development of countermeasures against COVID-19.

## Supplementary Materials

science.sciencemag.org/content/369/6511/1603/suppl/DC1 Materials and Methods Figs. S1 to S5 Tables S1 and S2

## Appendix

View/request a protocol for this paper from Bio-protocol.
